# Army and Navy Notes

**Published:** 1898-05

**Authors:** 


### ARMY AND NAVY NOTES.

It is announced that the secretary of war has made an allotment to the surgeon-general of the army of $20,000 out of the $50,000,000 emergency appropriation, to be used in the purchase of medical and hospital stores for the use of the army in the field in case of hostilities. The principal want of the medical department during extensive field operations is hospital stores, such as cots, blankets and ambulances. It is stated that the war department made an allotment on the 16th of April of $25,000 for the complete equipment of an army hospital at Key West.

Surgeon-General Sternberg, of the army, is negotiating for a large merchant vessel to be used as a floating army hospital to be stationed in the harbor selected as the base of supplies for the army operating against Cuba. The vessel is to be equipped with 500 beds and many of the contrivances now being installed in the naval ambulance ship Solace. Unlike the Solace, however, she will be stationary most of the time, except when it may be necessary to transport the sick and wounded to American ports. The Fall River line has offered its steamer Providence in this capacity.

A bill was recently introduced into the house of representatives providing for the appointment of fifteen additional assistant surgeons to the army with the rank of first-lieutenant. The bill further authorises the surgeon-general to appoint as many assistants in case of war as he deems necessary, the salary of each not to exceed $150 a month.

It has been asserted that the navy department would not accept the services of women as nurses. In contradiction of this statement, it is announced that surgeon-general Van Reypen has accepted the proffer of the services of twenty nurses offered by the Graduate Nurses Protective association, a regularly incorporated body of the state of New York. It is understood that these nurses will be assigned to duty on board the hospital steamer Creole, renamed the Solace, recently purchased of the Cromwell line. The uniform of these nurses will consist of a navy blue India silk waist and skirt with a white apron.

---

THE BOARD of naval inspectors at the naval hospital, Brooklyn, has received a large number of applications for the vacant places as acting assistant surgeon, of which there are 26. About 600 men have applied for places and as soon as the bill now before congress authorising the increase in the force becomes a law, the examinations will begin.

---

COL. C. R. GREENLEAF, Assistant Surgeon-General U. S. Army, lately stationed at San Francisco, has been ordered to Washington for duty on the staff of General Miles.

---

SURGEON-GENERAL STERNBERG has had applications for appointment from over 1,200 persons in the medical profession. The Surgeon-General, however, thinks the regular army medical corps is adequate for all present purposes. In the event of the occupation of Cuba, the department will endeavor to obtain physicians who are immune to yellow fever.

---

LIEUTENANT McKETHAN has finally been ordered to the command of the naval ambulance ship Solace. It has been difficult to obtain a commander for her as officers were anxious to serve in a more active capacity. Nevertheless, Mr. McKethan may find that he is serving his country quite as honorably on board the Solace as he could elsewhere. She was expected to take on stores at Norfolk, and after inspection by Surgeon-general Van Reypen to sail Wednesday, April 27th, for Admiral Sampson's fleet. The following medical officers have been ordered to the Solace: Passed Assistant Surgeons, B. T. Smith, C. F. Stokes, George Tucker Smith and Edward S. Bogert, Jr. They will be under the charge of Surgeon Thomas Streets.

